# Obese adolescents exhibit a constant ratio of GH isoforms after whole body vibration and maximal voluntary contractions

**DOI:** 10.1186/s12902-018-0323-6

**Published:** 2018-12-27

**Authors:** A. E. Rigamonti, M. Haenelt, M. Bidlingmaier, A. De Col, S. Tamini, G. Tringali, R. De Micheli, L. Abbruzzese, C. R. Goncalves da Cruz, M. Bernardo-Filho, S. G. Cella, A. Sartorio

**Affiliations:** 1 Department of Clinical Sciences and Community Health, University of Milan, Milan, Italy; 2 Endocrine Research Laboratories, Medizinische Klinik und Poliklinik IV, Klinikum der Universität München, Munich, Germany; 3 Istituto Auxologico Italiano, IRCCS, Experimental Laboratory for Auxo-endocrinological Research, Milan and Verbania, Italy; 4 Istituto Auxologico Italiano, IRCCS, Division of Auxology and Metabolic Diseases, Verbania, Italy; 5 Departamento de Biofisica e Biometria, Laboratório de Vibrações Mecânicas e Praticas Integrativas, Instituto de Biologia Roberto Alcantara Gomes, Universidade do Estado do Rio de Janeiro, Rio de Janeiro, Brazil; 6 Programa de Pós-Graduação em Ciências Médicas, Faculdade de Ciências Médicas, Universidade do Estado do Rio de Janeiro, Rio de Janeiro, Brazil

**Keywords:** Whole body vibration, Maximal voluntary contractions, GH isoforms, Obesity

## Abstract

**Background:**

Growth hormone (GH) is a heterogeneous protein composed of several molecular isoforms, the most abundant ones being the 22 kDa- and 20 kDa-GH. Exercise-induced secretion of GH isoforms has been extensively investigated in normal-weight individuals due to antidoping purposes, particularly recombinant human GH (rhGH) abuse. On the other hand, the evaluation of exercise-induced responses in GH isoforms has never been performed in obese subjects.

**Methods:**

The acute effects of whole body vibration (WBV) or maximal voluntary contraction (MVC) alone and the combination of MVC with WBV (MVC + WBV) on circulating levels of 22 kDa- and 20 kDa-GH were evaluated in 8 obese male adolescents [mean age ± SD: 17.1 ± 3.3 yrs.; weight: 107.4 ± 17.8 kg; body mass index (BMI): 36.5 ± 6.6 kg/m^2^; BMI standard deviation score (SDS): 3.1 ± 0.6].

**Results:**

MVC (alone or combined with WBV) significantly stimulated 22 kDa- and 20 kDa-GH secretion, while WBV alone was ineffective. In particular, 22 kDa- and 20 kDa-GH peaks were significantly higher after MVC + WBV and MVC than WBV. In addition, 22 kDa-GH (but not 20 kDa-GH) peak was significantly higher after MVC + WBV than MVC. Importantly, the ratio of circulating levels of 22 kDa- to 20 kDa-GH was constant throughout the time window of evaluation after exercise and similar among the three different protocols of exercise.

**Conclusions:**

The results of the present study confirm the ability of MVC, alone and in combination with WBV, to stimulate both 22 kDa- and 20 kDa-GH secretion in obese patients, these responses being related to the exercise workload. Since the ratio of 22 kDa- to 20 kDa-GH is constant after exercise and independent from the protocols of exercise as in normal-weight subjects, hyposomatotropism in obesity does not seem to depend on an unbalance of circulating GH isoforms. Since the present study was carried out in a small cohort of obese sedentary adolescents, these preliminary results should be confirmed in further future studies enrolling overweight/obese subjects with a wider age range.

## Background

Pituitary and circulating growth hormone (GH) is a heterogeneous mix of molecules deriving from different gene expression, alternative splicing, post-translational modifications, homo- or hetero-dimerization and oligomerization, peripheral metabolism and clearance. The predominant isoform is the 1- to 191-amino acid 22 kDa-GH, while alternative splicing of the exon 3 removes the residues 32–46 from the primary structure, yielding the 176-amino acid 20 kDa-GH, which represents about 15–20% of total circulating GH [1, 2].

In the last decade. Analytical methods have been implemented to measure circulating levels of GH isoforms with adequate accuracy, specificity and sensitivity [3].

Since the biochemical heterogeneity of GH implies a different binding of the ligand to the receptor, dimerization of GH receptor, receptor activation and signal transduction [2], measurement of GH isoforms is fundamental to understand the complexity of the physiological effects of GH or, more appropriately, “somatotropic hormones”.

Exercise-induced GH secretion has been extensively investigated in the last decades, several factors being identified to affect this response, including duration, intensity and protocol of exercise, training, nutritional status, body composition, age and gender of individuals undergoing exercise and concomitant pathophysiological conditions, such as GH deficiency (GHD) [4].

In this context, morbid obesity is characterized by hyposomatotropism, which is associated with blunted GH responses to a variety of pharmacological and physiological stimuli, including exercise [5, 6].

The interest of measuring circulating levels of GH isoforms after exercise has derived from the need to validate a promising antidoping test, based on the altered ratio of circulating GH isoforms in athletes administered with recombinant human GH (rhGH), which corresponds uniquely to 22 kDa-GH [7–9].

Some studies, carried out in normal-weight subjects, have revealed a post-exercise increase in circulating levels of GH isoforms, mainly 22 kDa- and 20 kDa-GH, being those of non-22 kDa-GH isoforms more sustained in the recovery phase, presumably for their long half-life [10–14]. Since non-22 kDa-GH isoforms are more hyperglycemic than 22 kDa-GH [15], the subject should be protected by post-exercise hypoglycaemia.

Surprisingly, to the best of our knowledge, no one has ever evaluated exercise-induced release of GH isoforms in obese subjects. Therefore, aim of the present study was to measure circulating levels of 22 kDa-GH and 20 kDa-GH in a cohort of obese subjects undergoing different protocols of exercise at increasing intensity (i.e. whole body vibration (WBV) and maximal voluntary contractions (MVC)]. For our experience, these types of exercise have demonstrated to be capable to stimulate “total” GH secretion in either normal-weight or obese subjects [16–20].

## Methods

Since the present study represents the development of a previous research performed in healthy normal-weight and obese subjects by our group, the methodology widely replicates that adopted in the previous papers [16, 18].

### Subjects

Eight obese male adolescents [mean age ± SD: 17.1 ± 3.3 years; weight: 107.4 ± 17.8 kg; body mass index (BMI): 36.5 ± 6.6 kg/m^2^; BMI standard deviation score (SDS): 3.1 ± 0.6; waist circumference: 118.3 ± 18.0 cm; hip circumference: 123.9 ± 15.3 cm] were recruited among patients hospitalized for a multidisciplinary integrated body weight reduction program at the Division of Auxology, Istituto Auxologico Italiano, Piancavallo (VB), Italy. In order to avoid any carry-over effect of the weight loss or modifications in diet and physical activity on GH secretion, the entire study was completed before starting the multidisciplinary integrated body weight reduction program.

All the adolescent patients voluntarily participated in this investigation after obtaining a written informed consent given by their parents. The main criteria of exclusion were any overt disease apart from morbid obesity. Diabetes mellitus, including glucose intolerance, was excluded by a 75 g oral glucose load, while Prader-Willi syndrome was excluded by clinical history, physical examination and FISH detection of chromosome 15 deletions. Due to the effects of female sex steroids on GH secretion and the difficulty to perform three exercise protocols (see below) in the same phase of the menstrual cycle, only boys were included in the study. Thyroidal (TSH and free T4 levels) and gonadal functions (LH, FSH, testosterone levels) were normal in all subjects (data not shown). All of them were habitually sedentary and were not involved in any strength or endurance training in the previous 2 weeks preceding the admission to the study protocol. No subjects had any signs of musculoskeletal disorders potentially hampering the execution of the tests.

No drugs or nutritional supplements known to interfere with GH and/or cortisol secretion were taken by the subjects in the month preceding the study.

### Testing

After an overnight fast, subjects were admitted to the laboratory 1 h before the beginning of the tests.

All the participants performed one of the following exercise protocols per diem: WBV alone, MVC alone, and MVC alternated with WBV (MVC + WBV).

The three different exercise protocols were randomly carried out in separate days with an interval of at least two days in between, accordingly with a scheme of randomization generated by the web-site https://www.randomizer.org.

**Fig. 1:**
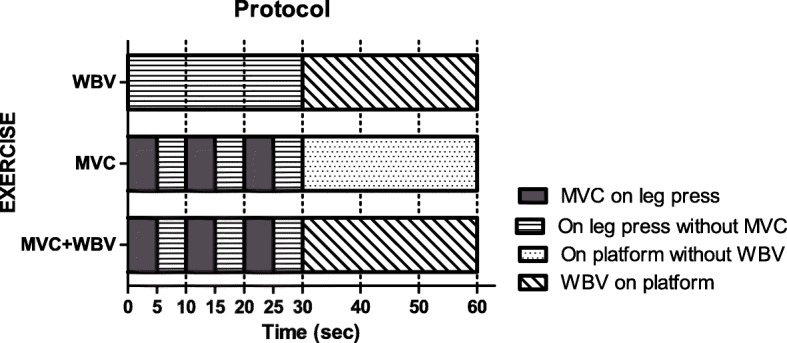
Schemes of protocols used in the study: whole body vibration only (WBV), maximal voluntary contractions only (MVC) and combination of maximal voluntary contractions and whole body vibration (MCV + WBV). See the text for further details

After a 5-min standardized warm-up on a cycloergometer (power: 50 W, cadence: 60 rpm), the three different protocols were performed as reported in Fig. 1.

During the WBV protocol, subjects initially seated in a semi-recumbent position on a horizontal leg press machine (Technogym, Gambettola, Italy) for 30 s, with the trunk-thigh and thigh-shank angles at 80°, without producing any movement. This first step was then followed by a 30-s WBV bout administered while the subject stood on a vibrating platform (Nevisys H1©, RME, Ferrara, Italy), as previously described [16]. Vertical sinusoidal vibrations were generated at a frequency of 35 Hz, the acceleration of the platform being 2.85 g at a peak-to-peak amplitude of vibration of 5 mm. These two rest-WBV cycles were repeated 15 times, resulting in a total duration of 15 min.

During the MVC protocol, subjects were initially placed on the leg press machine in the same supine position as described in the WBV protocol. The subjects performed three 5-s MVC, separated by 5-s resting periods in between. This first step was then followed by a 30 s of rest in the same static position as in the WBV protocol, but without WBV. These two MVC-rest cycles were repeated 15 times, resulting in a total duration of 15 min.

During the MVC + WBV protocol, subjects initially performed the three 5-s MVC (separated by 5-s resting periods in between) as in the MVC protocol. This first step was then followed by a 30-s WBV bout as in the WBV protocol. These two MVC + WBV cycles were repeated 15 times, resulting in a total duration of 15 min.

The characteristics of the different protocols, including the temporal patterns, are identical to those adopted in a previous work performed by our group [18] in healthy normal-weight subjects.

### Blood sampling and measurements of GH isoforms

Blood samples (5 ml at each time point) for measurement of 22 kDa- and 20 kDa-GH levels were drawn before the start of the experiment (baseline), immediately at the end of the exercise (T0) and after 15 (T15), 30 (T30), 45 (T45) and 75 min (T75). All blood samples were drawn through an indwelling cannula inserted into an ante-cubital vein kept patent via a continuous infusion of isotonic saline.

All blood samples were allowed to clot, centrifuged for 5 min and immediately stored at − 20 °C for the next analysis.

Serum levels of 22 kDa-GH were measured using the automated IDS iSYS hGH chemiluminescence assay system. In this assay, the detection monoclonal antibody (mAb) targets an epitope in the loop connecting helix 1 and 2 of GH, which is missing in 20 kDa-GH, thereby conferring specificity of the assay for the 22kD-GH molecules. Further details concerning the methodology had been published in a previous work [21]. In our hands, functional sensitivity of the assay was 0.04 μg/L and intra- and inter-assay coefficients of variation (CVs) both were below 5%.

Serum levels of 20 kDa-GH were measured using an in-house time resolved fluorescence assay as described before [22]. The assay employs two mAbs with no cross-reactivity to 22-kDa GH; intra- and inter-assay CVs were 5.4 and 6.3% at 0.2 μg/L and the functional sensitivity was 0.025 μg/L.

All samples were run in the same assay to minimize inter-assay variability (for both GH isoforms). Some samples fell below the limits of detection of 20 kDa-GH assay and were excluded from statistical analysis.

### Statistical analyses

The Sigma Stat 3.5 statistical software package was used for data analyses. GraphPad Prisma 5.0 software was used for plotting data.

In order to determine a priori the sample size, a power analysis was performed by considering a difference in the mean 22 kDa-GH levels at T0 between MVC + WVB and WBV equal to 2.5 ± 2.5 μg/L with an α error of 0.05 at two tails and a power of 0.80. The result of this calculation was *n* = 8 patients.

The Shapiro-Wilk test showed that all parameters were normally distributed.

Results are reported as mean ± SD (standard deviation). The responses in 22 kDa- and 20 kDa-GH were evaluated as absolute values for each experimental group (MVC + WBV, MVC and WBV). Peak values of GH isoforms represent the maximal levels reached during each experimental session.

Circulating levels of 22 kDa- and 20 kDa-GH and the related ratio (i.e., 22 kDa-GH/20 kDa-GH) were compared within each experimental group (vs. the corresponding basal value for MVC + WBV, MVC or WBV) over sampling times (intra-group analysis) and among the three experimental groups (MVC + WBV vs. MVC or WBV and MVC vs. WBV) for any sampling time (inter-group analysis) by using two-way ANOVA with repeated measures (with the two factors time and group and the interaction time × group), followed by the post hoc Bonferroni’s test. One-way ANOVA, followed by the post hoc Bonferroni’s test, was used to compare 22 kDa- and 20 kDa-GH peaks and the related ratio (i.e., 22 kDa-GH-peak/20 kDa-GH-peak) among MVC + WBV, MVC and WBV groups.

A level of significance of *p* < 0.05 was used for all data analyses.

## Results

**Fig. 2:**
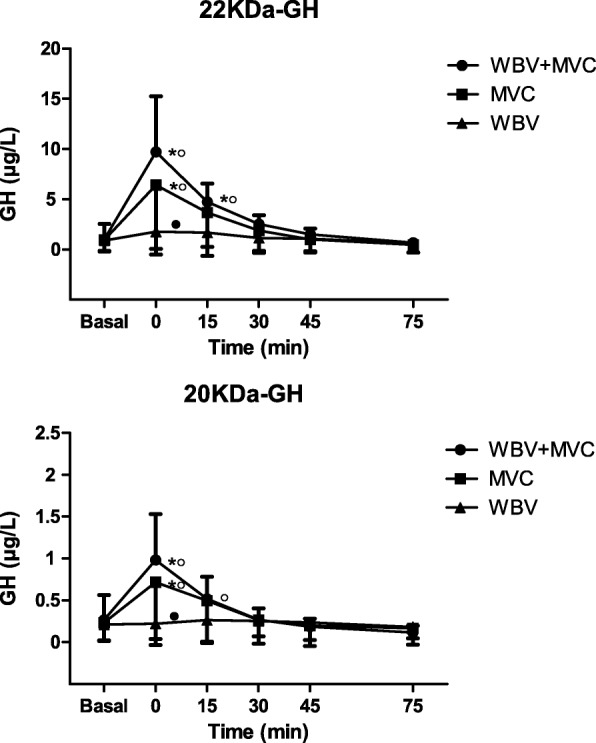
Circulating levels of 22 kDa- (top panel) and 20 kDa-GH (bottom panel) after the three different protocols of exercise: maximal voluntary contractions + whole body vibration (MVC + WBV), muscle voluntary contractions (MVC) and whole body vibration (WBV). Evaluation was performed at resting after the completion of the exercise. Values are expressed as mean ± SD. *p < 0.05 vs. the corresponding basal value (T0); ^o^*p* < 0.05 vs. the corresponding time point of WBV; ^●^*p* < 0.05 vs. the corresponding time point of MVC

**Fig. 3:**
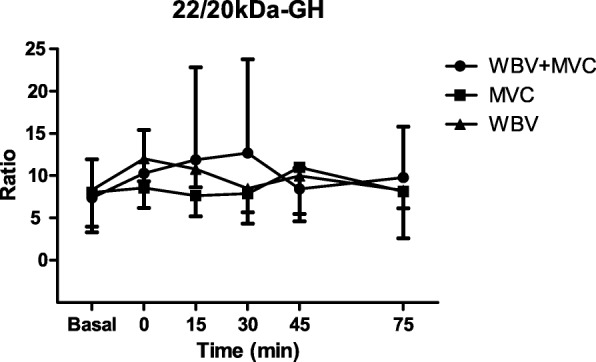
Ratio of circulating levels of 22 kDa-GH to 20 kDa-GH after the three different protocols of exercise: maximal voluntary contractions + whole body vibration (MVC + WBV), muscle voluntary contractions (MVC) and whole body vibration (WBV). Evaluation was performed at resting after the completion of the exercise. Values are expressed as mean ± SD

Mean basal circulating levels of 22 kDa- and 20 kDa-GH were 1.03 ± 1.52 μg/L and 0.269 ± 0.294 μg/L before MVC + WBV, 0.97 ± 1.15 μg/L and 0.234 ± 0.209 μg/L before MVC and 0.91 ± 1.05 μg/L and 0.212 ± 0.195 μg/L before WBV, respectively, without any significant difference (Fig. 2). The ratio of circulating levels of 22 kDa- to 20 kDa-GH at 0 min was similar among the three protocols of exercise (basal values: 7.382 ± 4.587 for MCV + WBV, 8.02 ± 4.73 for MCV and 8.31 ± 4.34, respectively) (Fig. 3).

MVC + WBV and MCV evoked a significant increase in 22 kDa-GH levels in obese patients at 0 and 15 min and only at 0 min vs. basal level, respectively (*p* < 0.05). WBV was ineffective to stimulate any 22 kDa-GH secretion. The 22 kDa-GH response was significantly higher after MVC + WBV than MVC at 0 min or WBV at 0 and 15 min and after MVC than WBV at 0 min (*p* < 0.05) (Fig. 2).

Similarly, MVC + WBV and MCV evoked a significant increase in 20 kDa-GH levels at 0 min vs. basal level (*p* < 0.05), without any significant changes after WBV. This response was significantly higher after MVC + WBV than MVC at 0 min or WBV at 0 and 15 min and after MVC than WBV at 0 min (p < 0.05) (Fig. 2).

**Fig. 4:**
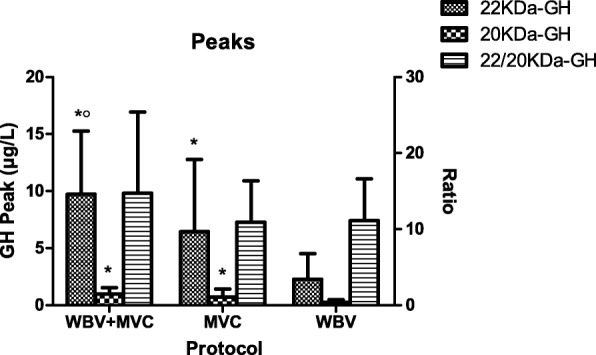
Peak values of 22 kDa- and 20 kDa-GH and the related ratio (i.e., 22 kDa-GH-peak/20 kDa-GH-peak) after the three different protocols of exercise: maximal voluntary contractions + whole body vibration (MVC + WBV), muscle voluntary contractions (MVC) and whole body vibration (WBV). Values are expressed as mean ± SD. *p < 0.05 vs. WBV; ^o^*p* < 0.05 vs. MVC

GH peaks after MVC + WBV (22 kDa-GH: 9.72 ± 5.55 μg/L and 20 kDa-GH: 0.98 ± 0.55 μg/L) and after MVC (22 kDa-GH: 6.45 ± 6.35 μg/L and 20 kDa-GH: 0.72 ± 0.68 μg/L) were significantly higher than those observed after WBV (22 kDa-GH: 2.27 ± 2.26 μg/L and 20 kDa-GH: 0.24 ± 0.25 μg/L) (*p* < 0.05), being only 22 kDa-GH peak after MVC + WBV higher than that after MCV (*p* < 0.05) (Fig. 4).

Importantly, the ratio of circulating levels of 22 kDa- to 20 kDa-GH in obese patients remained constant throughout the time window of evaluation and paralleled among the three protocols of exercises (Fig. 3). No significant differences were found in the ratio of peak levels of 22 kDa- to 20 kDa-GH among the three protocols of exercise (Fig. 4).

## Discussion

The most important findings of the present study carried out in a cohort of obese adolescents, generally more responsive to pharmacological or physiological GH stimuli than obese adults, were: (1) circulating levels of 22 kDa- and 20 kDa-GH are increased after acute exercise; (2) 22 kDa-GH is quantitatively the main isoform secreted in circulation after exercise; (3) combination of WBV with MVC evokes the highest responses in both GH isoforms; (4) the ratio of circulating levels of 22 kDa- to 20 kDa-GH is constant throughout the time window of evaluation after exercise and is similar among three different protocols of exercise (MVC + WBV, MVC and WBV).

Reportedly, the pathophysiology of hyposomatropism in obese subjects is complex, being presumably multifactorial. In particular, central (such as reduced GHRH function and/or increased somatostatin tone at the hypothalamic level) and peripheral (such as increased circulating levels of free fat acids, FFA) mechanisms have been invoked to explain the blunted GH responses to a variety of pharmacological and physiological stimuli in this special population [23–26]. Some authors have (erroneously) hypothesized the existence of altered proportions of GH isoforms in syndromic obesity (e.g., Prader-Willi syndrome) [27]. Now, we can exclude this possibility also in morbid obesity, being this the first study evaluating exercise-induced secretion of GH isoforms in an obese population and demonstrating a ratio of circulating levels of 22 kDa- to 20 kDa-GH similar to that of normal-weight individuals (i.e., about 10–15) [28]. Therefore, hyposomatotropism in morbid obesity does not derive from an alteration in the molecular mechanisms underlying the generation of GH isoforms, but seems to be a consequence of an insufficient stimulation and/or excessive inhibition of GH function (at hypothalamic and/or pituitary level). Obese state causes a “quantitative” rather than a “qualitative” alteration of GH secretion.

As shown in the present study, different protocols of exercise not surprisingly evoked responses in GH isoforms that were higher when the workload was heavier, such as that of MVC + WBV. This finding means not only the preservation of a “adequate” GH responsiveness to increasing physiological stimuli in obese subjects, but also the maintenance of a constant ratio of circulating levels of 22 kDa- to 20 kDa-GH independently from intensity, duration and protocol of exercise in both normal-weight and obese individuals.

In this context, the reliability of the antidoping test, used to detect rhGH abuse and based on an unbalance of circulating GH isoforms (particularly, the ratio of 22 kDa- tp pituitary GH, i.e. rGH/pitGH) after rhGH administration, would be ensured also in athletes, who, in some sport disciplines, are frequently overweight or obese [7, 9, 29]. In fact, as specified above the hyposomatotropism, acute exercise and execution of different protocols of exercise do not change the proportions of GH isoforms, which are similar to those present in normal-weight individuals 10,11,28]. Nevertheless, before extrapolating these data in a normative setting (e.g., WADA policy) [30], further studies in a larger population of overweight/obese athletes are mandatory, being the latter population different from ours, i.e., a small cohort of obese sedentary subjects.

A limitation of the present study could be the measurement of circulating levels of few GH isoforms, i.e. uniquely 22 kDa- and 20 kDa-GH, which have similar disappearance rates in circulation [10]. Since other GH isoforms, such as non-22 kDa-GH, persist in the recovery phase after acute exercise administered to normal-weight subjects [10], future studies should investigate the post-exercise kinetics of the remaining GH isoforms also in obese individuals. This issue is of great interest, being some GH isoforms more hyperglycemic than others [15] and exercise a well-recognized strategy to improve metabolic control [31]. In this respect, we cannot exclude that obese subjects have slower mechanisms of clearance for some GH isoforms, other than 22 kDa- and 20 kDa-GH, particularly those of higher molecular weight (e.g., dimers or oligomers).

The importance of measuring “all” GH isoforms is evident when comparing exercise-induced GH responses in studies in which different analytical methods were used. In particular, Rigamonti et al. [16] showed that WBV evoked a significant increase in (total?) GH levels, measured by a the commercially available immunometric chemiluminescence assay Immulite 2000 (DPC, Los Angeles, USA), while, in the present study, no significant changes in either 22 kDa- or 20 kDa-GH were found after WBV (alone). This might imply that some pharmacological and physiological stimuli selectively release some GH isoforms and not others or that, more likely, due to analytical reasons (e.g., sensitivity and specificity of the assays), GH stimulation may not be detectable and, erroneously, considered absent. So, before drawing any conclusion regarding the ineffectiveness of a GH stimulus, one should know what GH isoforms are measured. Since, as shown by recent biochemical and pharmacological studies [1, 2], activation of GH receptor depends on the specificity of GH isoform(s), measurement of (even very low) levels of any GH isoform is fundamental to understand signal transduction at molecular level and clinical effects in the endocrinological practice.

Finally, it is also worth mentioning that, in the present study, there was an increasing variability of circulating levels of GH isoforms during the recovery phase (i.e. starting from T30). This finding, already evidenced by Wallace et al. [10], is likely to be related to generation of GH fragments and/or dimers/oligomers in circulation, which could impair the specificity of our assays. However, despite this (potential) analytical interference, the ratio of circulating levels of 22 kDa- to 20 kDa is robustly stable.

Before closing, two other limitations should be recalled, i.e., the reduced number of subjects recruited and inclusion of only males. Therefore, the conclusions of the present study can be considered only preliminary; furthermore, we do propose a more extensive use of advanced analytical methods to measure GH isoforms in obesity and other pathophysiological conditions for better understanding the complexity of GH-IGF-I axis.

## Conclusions

The results of the present study confirm the ability of MVC, alone and in combination with WBV, to stimulate both 22 kDa- and 20 kDa-GH secretion in obese patients, being these responses related to the exercise workload. Since the ratio of 22 kDa- to 20 kDa-GH is constant after exercise and independent from the protocols of exercise as in normal-weight subjects, hyposomatotropism in obesity does not seem to depend on an unbalance of circulating GH isoforms and exercise-induced GH response is unlikely to affect the reliability of direct methods for detection of rhGH abuse in overweight/obese athletes. Since the present study was carried out in a small cohort of obese sedentary adolescents, these preliminary results should be confirmed by further future studies enrolling overweight/obese subjects with a wider age range.

## Abbreviations

BMI: Body mass index
FFA: Free fat acids
GH: Growth hormone
GHD: GH deficiency
GHRH: GH releasing hormone
IGF-I: Insulin-like growth factor I
MVC: Maximal voluntary contractions
rhGH: Recombinant human GH
SDS: Standard deviation score
WBV: Whole body vibration
